# Editorial: Modularity in motor control: from muscle synergies to cognitive action representation

**DOI:** 10.3389/fncom.2015.00126

**Published:** 2015-10-09

**Authors:** Andrea d'Avella, Martin Giese, Yuri P. Ivanenko, Thomas Schack, Tamar Flash

**Affiliations:** ^1^Department of Biomedical Sciences and Morphological and Functional Images, University of MessinaMessina, Italy; ^2^Laboratory of Neuromotor Physiology, Santa Lucia FoundationRome, Italy; ^3^Section for Computational Sensomotorics, Department of Cognitive Neurology, Hertie Institute for Clinical Brain Research and Center for Integrative Neuroscience, University Clinic TuebingenTuebingen, Germany; ^4^Research Group Neurocognition and Action-Biomechanics and Cognitive Interaction Technology-Center of Excellence, Bielefeld UniversityBielefeld, Germany; ^5^Department of Computer Science and Applied Mathematics, Weizmann Institute of ScienceRehovot, Israel

**Keywords:** modularity, motor control, muscle synergies, motor primitives, compositionality, action representation, robotics

Mastering a rich repertoire of motor behaviors, as humans and other animals do, is a surprising and still a poorly understood outcome of evolution, development, and learning. Many degrees-of-freedom, non-linear dynamics, and sensory delays provide formidable challenges for controlling even simple actions. Modularity as a functional element, both structural and computational, of a control architecture might be the key organizational principle that the central nervous system employs for achieving versatility and adaptability in motor control. Recent investigations of muscle synergies, motor primitives, compositionality, basic action concepts, and related work in machine learning have contributed, at different levels, to advance our understanding of the modular architecture underlying rich motor behaviors.

However, the existence and nature of the modules comprising the control architecture is far from settled. For instance, regularity and low-dimensionality of the motor output are often taken as an indication of modularity but they could simply be a byproduct of optimization and task constraints. Moreover, what are the relationships between modules at different levels, such as muscle synergies, kinematic invariants, and basic action concepts?

One important reason for the new interest in understanding modularity in motor control from different perspectives is the impressive development in cognitive robotics. In comparison to animals and humans, the motor skills of today's best robots are limited and inflexible. However, robot technology is maturing to the point at which it can start approximating a reasonable spectrum of different perceptual, cognitive, and motor capabilities. These advances allow researchers to explore how these motor, sensory, and cognitive functions might be integrated into meaningful architectures and to test their functional limits. Such systems provide a new test bed to explore different concepts of modularity and to experimentally investigate possible interactions between motor and cognitive processes.

Thus, the goal of this Research Topic is to review, compare, and debate theoretical and experimental studies of the modular organization of the motor control system at different levels. By bringing together researchers seeking to understand the building blocks of coordinating many muscles, planning endpoint and joint trajectories, and representing motor and behavioral actions in memory we aim at promoting new interactions between often disconnected research areas and approaches and providing a broad perspective on the notion of modularity in motor control.

## Reviews and perspectives

A number of review articles present and discuss available evidence, conceptual frameworks, and fundamental questions concerning modularity in motor control. These cover a range of issues such as the effective dimensionality, movement invariants, neural underpinnings, evolution, motor learning, and recovery of motor function.

Lacquaniti et al. (2013) provides a comprehensive review of evolutionary and developmental modules. These authors focus on modular control of locomotion to argue that the building blocks used to construct different locomotor behaviors are similar across several animal species, presumably related to ancestral neural networks of command. The authors present evidence that modular units of development are highly preserved and recombined during evolution.

In a thought-provoking review article, Duysens et al. (2013) argue that there is large overlap between the notions on modules and the older concepts of reflexes. They reason that facilitation of the flexor synergy at the end of the stance phase is linked to the activation of circuitry that is responsible for the generation of locomotor patterns (CPG, “central pattern generator”). More specifically, it is suggested that the responses in that period relate to the activation of a flexor burst generator. The latter structure forms the core of a new asymmetric model of the CPG. Beloozerova et al. (2013) review data on the differential controls for the shoulder, elbow, and wrist that are used by populations of neurons in the thalamo-cortical network. It is one of manifestations of a modular organization of control for locomotion. The authors hypothesize that this contributes to an effective control of a global limb parameter, the length of the stride, which results in a great reduction in variability of paw placement during accurate stepping.

Santello et al. (2013) propose a theoretical framework to reconcile important and still debated concepts such as the definitions of “fixed” vs. “flexible” synergies and mechanisms underlying the combination of synergies for hand control. d'Avella and Lacquaniti (2013) review recent results from the analysis of reaching muscle patterns supporting a control strategy consisted in the sequencing of time-varying muscle synergies. Alessandro et al. (2013b) review the works related to muscle synergies in neuroscience and control engineering and provide an overview of the methods that have been employed to test the validity of the control scheme. Specifically, the authors suggest that to assess the functional role of muscle synergies, synergy extraction methods should explicitly take into account task execution variables. Bizzi and Cheung (2013) address two critical questions: the explicit encoding of muscle synergies in the nervous system, and how muscle synergies simplify movement production and motor learning.

Another important field of research is the outcome of interventions in neurological disorders with motor deficits. Uncovering a common underlying neural framework for the modular control of movements and its dysfunction represents an interesting avenue for future work. Casadio et al. (2013) review the state of the art of computational models for neuromotor recovery from stroke through exercise, and their implications for treatment. The review specifically covers models of recovery at central, functional and muscle synergy level. Ivanenko et al. (2013) review various examples of adaptation of locomotor patterns in patients and discuss the findings in a general context of compensatory gait mechanisms, spatiotemporal architecture, and modularity of the locomotor program. Such investigations may have important implications related to the construction of gait rehabilitation technology. Further research needs to clarify whether plasticity in muscle patterns originates from sharing common modules or by creating new muscle synergies and whether the rehabilitation programs may benefit from revitalizing the modules underlying motor behaviors.

## Muscle synergies

Amongst the original research articles, a large group of contributions is dedicated to the modular organization of multi-muscle activity across different motor tasks. It has been hypothesized that the nervous system simplifies muscle control through modularity, using neural patterns to activate muscles in groups called synergies.

An important example of ongoing debate is the current discussion of the critical aspects and organization of muscle synergies. de Rugy et al. (2013) argue that the usefulness of muscle synergies as a control principle should be evaluated in terms of errors produced and, using data from a force-aiming task in two dimensions, illustrate through simulation how synergy decomposition inevitably introduces substantial task space errors. They also show that the number of synergies required to approximate the optimal muscle pattern for an arbitrary biomechanical system increases with task-space dimensionality, which indicates that the capacity of synergy decomposition to explain behavior depends critically on the scope of the original database. Steele et al. (2013) present evidence that the number and choice of muscles impact the results of muscle synergy analyses. Thus, researchers should be cautious in evaluating muscle synergies when EMG is measured from a small subset of muscles.

Delis et al. (2013a,b) stress the effectiveness of the decoding metric in systematically assessing muscle synergy decompositions in task space and the functional role of trial-to-trial correlations between synergy activations. The results of Chiovetto et al. (2013) support the notion that each EMG decomposition provides a set of well-interpretable muscle synergies, identifying reduction of dimensionality in different aspects of the movements. Borzelli et al. (2013) test whether the CNS generates forces by minimum effort recruitment of either individual muscles or muscle synergies during the generation of isometric forces at the hand. The minimum effort recruitment of synergies predicts the observed muscle patterns better than the minimum effort recruitment of individual muscles. Russo et al. (2014) compare the torques acting at four arm joints during fast reaching movements in different directions and show that muscle pattern dimensionalities are higher than torques dimensionalities. They argue that this is necessary to overcome the non-linearities of the musculoskeletal system and to flexibly generate endpoint trajectories with simple kinematic features using a limited number of building blocks.

In the context of direction-specific recruitment of muscle synergies, Gentner et al. (2013) investigate adaptation to a visuomotor rotation of a virtual target displacement and show that the structure of muscle synergies is preserved, suggesting that changes in muscle patterns are obtained by rotating the directional tuning of the synergy recruitment. Bengoetxea et al. (2014a,b) employ a dynamic recurrent neural network (DRNN) and principal component analysis of EMG activity during discrete and rhythmic arm movements. The authors discuss consistent patterns of muscle groupings in the context of their functional organization for controlling orthogonal movement directions. Berger and d'Avella (2014) recorded EMG activity and isometric hand forces during a force-aiming task in a virtual environment. In contrast to de Rugy et al. (2013), they show that muscle synergies can be used to generate target forces in multiple directions with the same accuracy achieved using individual muscles. Strikingly, human subjects are able to perform the task immediately after switching from force-control to EMG-control and synergy-control, suggesting that muscle synergies provide an effective strategy for motor coordination.

Whether muscle synergies are shared across tasks or they are task-specific is another debated aspect of modularity. Chvatal and Ting (2013) compare muscle synergies during multidirectional support-surface perturbations during standing and walking, as well as unperturbed walking. They find both shared and task-specific muscle synergies, suggesting that differences in muscle synergies across conditions reflect differences in the biomechanical demands of the tasks and that muscle synergies may define a repertoire of biomechanical subtasks recruited according to task-level goals. Frere and Hug (2012) demonstrate that the muscle synergies are consistent across experienced gymnasts, even during a skilled motor task that requires learning. De Marchis et al. (2013) investigate muscle synergies during pedaling in humans. Additional modules are identified when visual feedback about mechanical effectiveness is available and the structure of the identified modules is found similar to that extracted in other studies of human walking, confirming the existence of shared and task specific muscle synergies. Finally, Hart and Giszter (2013) present a method that uses point process statistics to discriminate the forms of synergies in motor pattern data. According to this method, frog and rat EMG data are most consistent with synchronous synergy models, supporting separated control of rhythm and pattern of motor primitives.

## Motor primitives at the kinematic level

A number of contributions aim at understanding motor primitives at the kinematic level. Zelman et al. (2013) explore whether different octopus arm movements are built up of elementary kinematic units by decomposing surfaces, representing curvature, and torsion values of the paths of points along the arm, into a weighted combination of 2D Gaussian functions, considered as motion primitives at the kinematic level of octopus arm movements. Endres et al. (2013a) investigate the endpoint trajectories of human movements (sign language) that are characterized by the power laws linking velocity and curvature. The parameters of these power laws are exploited for the unsupervised segmentation of actions into movement primitives. Sternad et al. (2013) propose that control of sensorimotor behavior may utilize dynamic primitives. Their results clearly indicate a gradual transition between discrete and rhythmic arm movements, supporting the proposal that representation is based on primitives rather than on veridical internal models. Boyer et al. (2013) investigate interactions between the auditory and motor systems to uncover different modular neural processes involved in the multisensory and motor representations of targets in goal-directed movements and corresponding reference frames for each sensory modality. Racz and Valero-Cuevas (2013) suggest that the similar nature of control actions across time scales in both task-relevant and task-irrelevant spaces points to a level of modularity not previously recognized in motor tasks. Hogan and Sternad (2013) propose that the spectacular performance of a wide range of upper- and lower-limb behaviors arises from encoding motor commands in terms of three classes of dynamic primitives: submovements, oscillations, and mechanical impedances. They present some methods for addressing the challenges posed by the experimental identification of these dynamic primitives and consider the implications of this theoretical framework for locomotor rehabilitation.

## Neural substrates

Another exciting area explored in this Research Topic is potential neural substrates for modularity in motor control and action representation. Takei and Seki (2013) argue about synaptic and functional linkage between spinal interneurons and the organization of hand-muscle synergies. Abeles et al. (2013) discuss the compositional structure of hand movements by analyzing and modeling neural and behavioral data obtained from experiments where monkeys performed scribbling movements. A classification of the neural data employing a hidden Markov model shows a coincidence of the neural states with the behavioral categories of movement segmentations that are primarily parabolic in shape. Overduin et al. (2014) investigate whether muscle synergies evoked by intracortical microstimulation (ICMS) in rhesus macaques are similarly encoded by nearby motor cortical units during object reach, grasp, and carry movements. They find that the synergy most strongly evoked at an ICMS site matches the synergy most strongly encoded by proximal units more often than expected by chance. The results suggest a common neural substrate for microstimulation-evoked motor responses and for the generation of muscle patterns during natural behaviors. Krouchev and Drew (2013) describe a modular organization of the locomotor step cycle in the cat in which a number of sparse synergies are activated sequentially during unobstructed locomotion and during voluntary gait modifications. The authors argue that the changes in phase and magnitude of a finite number of muscle synergies could be produced by changes in the activity of neurons in the motor cortex. Mokienko et al. (2013) study motor imagery of grasping movements and corresponding neural underpinnings in brain-computer interface trained human subjects.

## Models

A number of modeling papers address different aspects of modularity. In a multi-directional reaching task simulated with a musculoskeletal model of the human arm, Ruckert and d'Avella (2013) propose a movement primitive representation that employs parametrized basis functions, which combines the benefits of muscle synergies and dynamic movement primitives, and show how movement primitives can be used to learn appropriate muscle excitation patterns and to generalize effectively to new reaching skills. Sartori et al. (2013) use a Gaussian-shaped impulsive excitation curves or primitives as input drive for large musculoskeletal models across different human locomotion tasks. Alessandro et al. (2013a) examine the feasibility of controlling non-linear dynamical systems by linear combinations of a small set of torque profiles or motor synergies and suggest that in order to realize an effective and low-dimensional controller, synergies should embed features of both the desired tasks and the system dynamics.

Significant progress has been made with respect to some fundamental questions concerning optimization of control architectures and motor learning. Rückert et al. (2012) propose a movement primitive representation based on probabilistic inference in learned graphical models with properties that comply with salient features of biological movement control. In simulations of a complex 4-link balancing task, they show that movement primitives facilitate learning and lead to better generalization. Endres et al. (2013b) address the issue of the selection of the parameters of movement primitive models or the model type and propose an approach based on a Laplace approximation to the posterior distribution of the parameters of a given blind source separation model. They validate the approach on simulated data and on human gait data, finding that an anechoic mixture model with a temporal smoothness constraint on the sources can best account for the data. Kuppuswamy and Harris (2014) investigate whether muscle synergies can reduce the state-space dimensionality while maintaining task control. Based on the observation that constraining the control input to a weighted combination of temporal muscle synergies also constrains the dynamic behavior of a system in a trajectory-specific manner, they show that smooth straight-line Cartesian trajectories with bell-shaped velocity profiles emerged as the optima for the reaching task and that trajectory and synergy specific dimensionality reduction results from muscle synergy control. Hayashibe and Shimoda (2014) aim at identifying a modular control architecture realizing adaptability and optimality without prior knowledge of system dynamics. They propose a novel motor control paradigm based on tacit learning with task space feedback. The proposed paradigm can optimize solutions for reaching with a three-joint, planar biomechanical model, acquiring motor synergy, and finding energy efficient solutions for different load conditions.

A few contributions further examine the usage of neural networks. Schilling et al. (2013) demonstrate a solution for the selection and sequencing of different (attractor) states required to control different behaviors of a hexapod walker as forward walking at different speeds, backward walking, as well as negotiation of tight curves. The proposed control architecture of a recurrent neural network is characterized by different types of modules being arranged in layers and columns, and can also be considered as a holistic system showing emergent properties which cannot be attributed to a specific module. Hoellinger et al. (2013) describe the use of a DRNN mimicking the natural oscillatory behavior of human locomotion for reproducing the planar covariation rule in both legs at different walking speeds. This emerging property in the artificial neural networks resonates with recent advances in neurophysiology of inhibitory neurons that are involved in central nervous system oscillatory activities. The main message of this study is that this type of DRNN may offer a useful model of physiological central pattern generators for the purpose of gaining insights in basic research and developing clinical applications.

Ehrenfeld et al. (2013) address the question of how the brain maintains a probabilistic body state estimate over time from a modeling perspective. The results showed that the neural estimates can detect and decrease the impact of false sensory information, can propagate conflicting information across modules, and can improve overall estimation accuracy due to additional module interactions. Finally, Tagliabue and Mcintyre (2014) review different formulations of concurrent models for sensory integration and propose a modular approach in which the overall behavior is built by computing multiple concurrent comparisons carried out simultaneously in a number of different reference frames.

## Robotics

Findings in biological research concerning a modular control hierarchy, which combines movement/motor primitives into complex and natural movements, inspire engineers in the quest for adaptive and skillful control for robots. Neumann et al. (2014) present a unified approach for learning a modular control architecture, introducing new policy search algorithms that are based on information-theoretic principles and are able to learn to select, adapt, and sequence the building blocks to compose more complex behaviors. The authors summarize their experiments for learning modular control architectures in simulation and with real robots. Waegeman et al. (2013) propose a modular architecture with control primitives (MACOP) which uses a set of controllers, where each controller becomes specialized in a subregion of its joint and task-space. The authors evaluate MACOP on a numerical model of a robot arm by training it to generate desired trajectories and show how MACOP compensates for the dynamic effects caused by a fixed control rate and the inertia of the robot. Nakajima et al. (2013) explore the idea that control, which is conventionally thought to be handled by the brain or a controller, can partially be outsourced to the physical body and the interaction with the environment. By using a soft robotic arm inspired by the octopus they show in a number of experiments how control is partially incorporated into the physical arm's dynamics and how the arm's dynamics can be exploited to approximate non-linear dynamical systems. Spröwitz et al. (2014) implement kinematic primitives for walking and trotting gaits of a quadruped robot and show that a very low complexity of modular, rhythmic, feed-forward motor control is sufficient for level-ground locomotion in combination with passive compliant legged hardware.

## Intermittent control

Evidence for intermittency in human motor control has been repeatedly observed in the neural control of movement literature and it has been discussed in this Research Topic in the context of the modular organization of the motor control system. Karniel (2013) focuses on an area in which intermittent control has not yet been thoroughly considered, with respect to the structure of muscle synergies. He presents the minimum transition hypothesis and its predictions with regard to the structure of muscle synergies. D'Andola et al. (2013) demonstrate that that the control of interceptive movements (catching a flying ball) relies on a combination of reactive and predictive processes through the intermittent recruitment of time-varying muscle synergies. van de Kamp et al. (2013) explore modular organization in whole body control architecture within the intermittent control paradigm with an intermittent interval of around 0.5 s. The authors suggest that parallel sensory input converges to a serial, single channel process involving planning, selection, and temporal inhibition of alternative responses prior to low dimensional motor output and may underlie the flexibility of human control. Such studies may have important implications with respect to the design of brain machine interfaces and human robot interaction.

## Action representation

The final theme we have identified in the contributions centers on the modular organization and interaction between motor and cognitive processes. Land et al. (2013) explore the links between cognitive and biomechanical levels of motor control in order to understand the extent to which the output at a kinematic level is governed by representations at a cognitive level of motor control. The authors apply a new spatio-temporal decomposition method for assessing memory structures underlying complex actions in order to investigate the overlap between the structure of motor representations in memory and their corresponding kinematic structures.

Taken together, this Research Topic demonstrates the impressive breadth of research currently being undertaken on modularity in motor control.

### Conflict of interest statement

The authors declare that the research was conducted in the absence of any commercial or financial relationships that could be construed as a potential conflict of interest.
